# Development and Application of an Indirect Enzyme‐Linked Immunosorbent Assay Based on a Recombinant Matrix Protein for the Serological Study of Porcine Deltacoronavirus in Mexican Pigs

**DOI:** 10.1002/vms3.70108

**Published:** 2024-11-04

**Authors:** Francisco Jesus Castañeda Montes, José Luis Cerriteño Sánchez, Julieta Sandra Cuevas‐Romero, María Azucena Castañeda Montes, Susana Mendoza Elvira

**Affiliations:** ^1^ Estancias Posdoctorales por México para la Formación y Consolidación de las y los Investigadores por México CONAHCYT Mexico City Mexico; ^2^ Centro Nacional de Investigación Disciplinaria en Salud Animal e Inocuidad Instituto Nacional de Investigaciones Forestales Agrícolas y Pecuarias Mexico City Mexico; ^3^ Dirección de Procesos Alimentarios y Química Área Biotecnología, Unidad Académica de Capulhuac Universidad Tecnológica del Valle de Toluca Lerma Mexico; ^4^ Laboratorio de Virología, Genética y Biología Molecular, Facultad de Educación Superior, Cuautitlán, Medicina Veterinaria Universidad Nacional Autónoma de México Cuautitlán Izcalli Estado de México Mexico; ^5^ Posgrado en Ciencias de la Producción y de la Salud Animal Facultad de Estudios Superiores, Universidad Nacional Autónoma de México Cuautitlan Estado de México Mexico

**Keywords:** indirect enzyme‐linked immunosorbent assay (iELISA), M protein, porcine deltacoronavirus, seroprevalence

## Abstract

Porcine deltacoronavirus (PDCoV) is an infectious disease that causes diarrhoea in pigs of different ages; however, piglets are more susceptible. PDCoV was first reported in 2012 in China and Hong Kong. Later, it was first reported in the USA in 2014 and in Mexico in 2019. Several studies have shown that M protein is highly conserved and, therefore, suitable for diagnostic systems. In this study, for the first time, an indirect enzyme‐linked immunosorbent assay (iELISA) based on a recombinant M protein (*r*M‐PDCoV) was developed to evaluate the seroprevalence of PDCoV in four states in Mexico. High sensitivity (83%) and specificity (100%) were observed for the iELISA. The kappa index calculated a nearly perfect agreement (0.8831) compared to the Western blot (gold standard test), suggesting acceptable statistical value support. In this study, 50.38% of the serum samples from backyard pigs were PDCoV‐positive. The serological comparison showed that PDCoV/PEDV coinfections occurred in 31.98% of the analysed sera. These results can enrich our understanding of how this virus spreads and enable the evaluation of PDCoV infections. Moreover, it highlights the importance of continually investigating the seroprevalence of PDCoV in Mexico because there is also no information about the current prevalence of the disease.

## Graphical abstract

Developed an iELISA with the PDCoV recombinant protein M to sero‐evaluate 516 samples from 4 different states of Mexico and serologically compare PDCoV/PEDV.

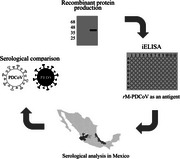

## 1 Introduction

Porcine deltacoronavirus (PDCoV) was first reported in 2012 in China and Hong Kong. The virus was found in pig faeces (Woo et al. 2012). In 2014, the Ohio Department of Agriculture first announced the presence of PDCoV in the USA. Two strains were identified: OH1987 and USA/IA/2014/8734 (Wang et al. 2014). Later, in 2019, PDCoV was first reported in Mexico, and it was determined that the Mexican isolates are closely related to the USA isolates (Perez‐Rivera et al. 2019). Subsequently, PDCoV has been detected in Canada, South Korea, Thailand, Vietnam, Laos, Taiwan, Japan and Haiti.

PDCoV is an enveloped single‐stranded positive‐sense RNA virus with a genome size of approximately 25.4 kb that encodes four structural proteins: spike (S), envelope (E), membrane (M) and nucleocapsid (N); and four nonstructural proteins (He et al. 2020). Several studies have shown that the PDCoV M protein is highly conserved (Castaneda‐Montes, Cerriteno‐Sanchez, et al. 2023; Wu et al. 2023; Zhang et al. 2012). On the other hand, the M protein can promote the production of antibodies with neutralizing activity (Weiss and Leibowitz 2011). The production of these antibodies has been observed in TGEV (Dong, Zhang, et al. 2016), PEDV (Zhang et al. 2012), SARS‐CoV (He et al. 2005; Liu et al. 2010; Qian et al. 2006), feline infectious peritonitis virus (FIPV) (Takano et al. 2014) and avian infectious bronchitis coronavirus (IBV) (Xing et al. 2009). Therefore, the PDCoV M protein could be used as an antigen in an indirect enzyme‐linked immunosorbent assay (iELISA) (Castaneda‐Montes, Cerriteno‐Sanchez, et al. 2023; Luo et al. 2017; Thachil et al. 2015)

PDCoV can cause diarrhoea in pigs of different ages. However, piglets are more susceptible (Feng, Xu, and Zhu 2020; Wang et al. 2014). It has been reported that the faecal–oral route may be the primary source of transmission of PDCoV. Once the virus enters and infects enterocytes, it rapidly leads to a villous atrophy in the small intestine (jejunum and ileum) (Hu et al. 2015). This viral infection causes a thin and transparent intestinal wall, causing the accumulation of a large amount of fluid in the intestinal lumen and occasionally coagulated milk in the stomach (Wu et al. 2019; Xu et al. 2018). The clinical signs are characterized by acute and severe watery diarrhoea, vomiting, dehydration, lethargy and anorexia (Chen et al. 2015; Dong, Fang, et al. 2016; Ma et al. 2015).

The clinical signs of PDCoV were reported to be less severe than those of PEDV infection. However, co‐infection with PEDV, TGEV and other enteric viruses like porcine rotavirus (PRV) is common and can lead to more serious clinical signs that are mainly characterized by longer diarrhoea periods (Marthaler et al. 2014; Saeng‐Chuto et al. 2021; Zhang et al. 2019; Zhang et al. 2022). It has been reported that the prevalence of PDCoV in pigs is about 30% (Marthaler et al. 2014; Wang et al. 2014; Woo et al. 2012). In 2019, a study carried out in Mexico showed that the most frequent PDCoV coinfection was PEDV with a rate of 54.1% (Perez‐Rivera et al. 2019). However, there is no thorough information about the seroprevalence of PDCoV or the distribution of this disease in Mexico. In the first seroepidemiologic study of PEDV in Mexico, it was reported that 61.66% of the serum samples were positive, showing that PEDV is still circulating in the main pig‐producing states in Mexico (Garcia‐Gonzalez et al. 2023). Therefore, PDCoV–PEDV coinfection is possible, with subsequent negative effects of these diseases.

The methods used to diagnose PDCoV can be divided into virological and serological methods. Virological methods include the detection of virus particles. Serological assays determine previous exposure by identifying the presence of antibodies (Zhang 2016). Serological tests based on the detection of immunoglobulin and antibodies in serum, colostrum and faecal samples have been widely used and accepted for swine enteric coronavirus (SeCoV) (Gerber et al. 2014; Gerber and Opriessnig 2015). However, there is no official method issued by a government health regulatory institute to determine the seroprevalence of PDCoV in Mexico, although a recombinant PDCoV antigen has already been developed and characterized with favourable results when it is incorporated into an ELISA assay. Thus, in this study, an iELISA, based on a previously developed recombinant M protein (Castaneda‐Montes, Cerriteno‐Sanchez, et al. 2023), was used to evaluate 516 pig sera from a slaughterhouse to provide information about the seroprevalence of the PDCoV in 4 states in Mexico: Aguascalientes, Guanajuato, Jalisco and Veracruz. Together, these states account for 40% of the total swine production in Mexico (https://www.gob.mx/siap/acciones‐y‐programas/panorama‐agroalimentario‐258035, accessed March 2024). The results can explain how the virus spreads and retrospectively evaluate the performance of the PDCoV infections.

## 2 Materials and Methods

### 2.1 Serum Samples

A total of 516 serum samples from 4 Mexican states were evaluated via iELISA with a recombinant PDCoV membrane protein (*r*M‐PDCoV) as an antigen. The serum was separated from blood samples via centrifugation at 1500 × *g* for 10 min and was preserved at −20°C until use. The 516 sera comprised 150 from Guanajuato, 186 from Aguascalientes, 134 from Jalisco and 46 from Veracruz. The serum samples were collected from 2019 to 2021 from ≥6‐month‐old backyard pigs and sows.

### 2.2 Production and Purification of *r*M‐PDCoV

A total of five batches of *r*M‐PDCoV were produced using the developed competent *Escherichia coli* BL21 cells as an expression system as previously reported by Castaneda‐Montes, Cerriteno‐Sanchez, et al. (2023). Briefly, the cells were grown in 200 ml of Luria Broth at 37°C and 250 rpm with 50 µg/µL of kanamycin. Later, when the optical density of the culture reached 0.5 (OD_600 nm_), 1.5 M of IPTG (Merck KGaA, Darmstadt, Germany) was added, and the culture was grown overnight at 37°C and 250 rpm. Then, induced cells were retrieved from the medium via centrifugation (4000 rpm for 10 min), and supernatants and pellets were mechanically homogenized using GAULIN at 800 kg/cm^3^ for 15 min. The *r*M‐PDCoV was analysed via 12% SDS–PAGE and visualized using Coomassie Brilliant Blue G‐250 and Western blotting (WB). *r*M‐PDCoV was purified using a HisTrap Chelating High Performance Ni‐NTA agarose column (5 mL) (GE Healthcare, Chicago, IL, USA). Using a 12% SDS–PAGE, the purified *r*M‐PDCoV was separated. It was later confirmed using WB (as mentioned above) to have an expected molecular weight of 37.3 kDa. The protein concentration was determined according to the Bradford method (Bradford 1976).

### 2.3 Development of the iELISA

The iELISA was carried out according to Castaneda‐Montes, Cerriteno‐Sanchez, et al. (2023). Briefly, a 96‐well microplate (Nalge Nunc International Corp., Rochester, NY, USA) was coated using different amounts of purified *r*M‐PDCoV per well in a 50 mM carbonate–bicarbonate buffer (pH = 9.6) with a final volume of 100 µL. The plate was then washed with PBS‐Tween 0.1% and blocked with PBS‐Tween (20 mM Tris–HCl, pH 8, 0.15 M NaCl and 0.05% Tween 20) with 5% skim milk (dilution buffer) for 1 h at 37°C. Serum samples were diluted in PBS‐Tween with 5% skim milk and the dilutions were evaluated. Later, 100 µL per well of the optimal serum dilution was incubated for 1 h at 37°C. Then, different dilutions were used (Table 1).

**TABLE 1:** Different conditions were evaluated for the indirect enzyme‐linked immunosorbent assay (iELISA) optimization.

Antigen (*r*M‐PDCoV)	25 ng/well	50 ng/well	75 ng/well
Serum sample dilution	1:500	1:1000	1:2000
Conjugated antibody dilutions	1:15,000	1:17,000	1:20,000

Abbreviation: PDCoV, porcine deltacoronavirus.

Anti‐pig‐HRP (Goat anti‐Pig IgG HRP Conjugate, ImmunoReagents Inc., USA) was incubated in a volume of 100 µL for 1 h at 37°C with slight agitation. The anti‐pig‐HRP dilution was used in WB according to the manufacturer instructions (1:500–1:5000). On the other hand, for ELISA, the optimal dilution was determined by our laboratory according to other publications (Castaneda‐Montes, Cuevas‐Romero, et al. 2023; Garcia‐Gonzalez et al. 2023; Lara‐Romero et al. 2022). Finally, the plate was washed with PBS‐Tween, and 100 µL of a 3,3′,5,5′‐tetramethylbenzidine (TMB) (SeraCare, Milford, MA, USA) substrate solution was added to each well and incubated at room temperature for 15 min. The reaction was stopped with 100 µL of 2 M sulphuric acid, and the optical density was measured at 450 nm (OD 450 nm).

### 2.4 Immunoreactivity of Swine Serum Samples to *r*M‐PDCoV by WB

In total, 30 negative and 30 positive pig serum samples previously tested via iELISA, as described above, were randomly selected to be confirmed using WB. These pig samples were used to establish the sensitivity and specificity of the developed iELISA. The WB using pig serum samples was made as follows: The serum samples were used as a primary antibody (1:500) and an HRP‐labelled anti‐pig antibody was used as a secondary antibody (1:5000). Both antibodies were diluted in 5% nonfat milk in a PBS‐Tween buffer. Briefly, 300 ng of purified *r*M‐PDCoV protein was electrotransferred onto a nitrocellulose membrane with a pore size of 0.45 µm (Advanced Microdevices Pvt. Ltd. India). The membranes were blocked with 5% nonfat milk in a TBS‐Tween buffer at 4°C for 1 h with gentle agitation. Protein bands were visualized with a DAB substrate (3,3′‐diaminobenzidine tetrahydrochloride) (Sigma‐Aldrich, St. Louis, MO, USA) with 10 mL of a substrate solution (PBS, 12 mg of DAB, and 300 µL 3.4% H_2_O_2_).

### 2.5 Sensitivity, Specificity and Concordance (*K*) of the iELISA Assay

The sensitivity and specificity values were calculated using a 2 × 2 contingency table (Table 2). Moreover, the contingency chart described above was used to determine the kappa index (*K*). *K* corresponds to the agreement of the test and ranges from 0 to 1, where 0 indicates no agreement with the WB (gold standard test) and 1 indicates perfect agreement (McHugh 2012).

**TABLE 2:** 2 × 2 contingency table used to determine the sensitivity and specificity values.

	PDCoV positive	PDCoV negative	Total
Positives serums	*a*	*b*	***r*** = *a* + *b*
Negative serums	*c*	*d*	***s*** = *c* + *d*
Total	*t* = *a* + *c*	*u* = *b* + *d*	***N*** = *a* + *b* + *c* + *d*

Abbreviation: PDCoV, porcine deltacoronavirus.

Furthermore, the percentages of sensitivity and specificity were calculated using

(1) $$\mathrm{Sensitivity}\;=\left[\;\frac{a}{a+c}\;\right]\;\times \;100$$

(2) $$\mathrm{Specificity}\;=\left[\;\frac{d}{b+d}\;\right]\;\times \;100$$

where *a* is the number of true positives; *b* is the number of false positives; *c* is the number of false negatives; and finally, *d* is the number of true negatives. Moreover, the *K* was calculated using

(3) $$K=Po-\frac{Pe}{1-Pe},\;\mathrm{where}:\;Po=\frac{\left(a+d\right)}{N}\;\mathrm{and}\;Pe=\frac{r+t+s+u}{N^{2}}$$

where the kappa index value ranges from 1 (complete agreement) to 0 (agreement is equal to that expected by chance). Values of *K* > 0.81 mean nearly perfect agreement; 0.61–0.80 indicate substantial agreement; 0.41–0.60 indicate moderate agreement; 0.21–0.40 indicate fair agreement; 0–0.20 indicate slight agreement; and 0 indicate poor agreement (McHugh 2012). *Po* is the proportion of agreements observed; *Pe* is the proportion of agreements expected in the hypothesis of independence; *r* is the sum value of *a* and *b*; *s* is the sum value of *c* and *d*; *t* is the sum value of *a* and *c*; *u* is the sum value of *b* and *d*; and *N* is the sum of *r*, s, *t* and *u*.

On the other hand, in this study, the cut‐off value was determined as ±3 standard deviations (OD_450 nm_). Positive and negative samples were selected above and below the cut‐off value, respectively.

### 2.6 Analysis of the Results (Percentage of Positivity Determination)

Using the optimal iELISA conditions, the 516 serum samples were analysed, and the serum results were expressed as the percentage of positivity (PP). The PP values were used to determine the acceptance of the test of serum absorbance for a diagnostic interpretation. The PP was calculated as follows:

(4) $$\mathrm{PP}\;=\left[\;\frac{\mathrm{RA}}{\mathrm{MA}}\;\right]\;\times \;100$$

where RA is the replicate of the absorbance value of the test serum, and MA is the median absorbance value of the positive control. Therefore, the absorbance of each tested sample was expressed as a percentage of a highly positive reference standard to overcome the relativity of the measured absorbance (Mozsik 2021). The cut‐off absorbance value was expressed as PP. The results were graphed using SigmaPlot version 12.5 (Systat Software Inc., San Jose, CA, USA). Maps were constructed using MapChart (https://www.mapchart.net/, accessed in March 2023).

## 3 Results

### 3.1 Production of *r*M‐PDCoV

**FIGURE 1:**
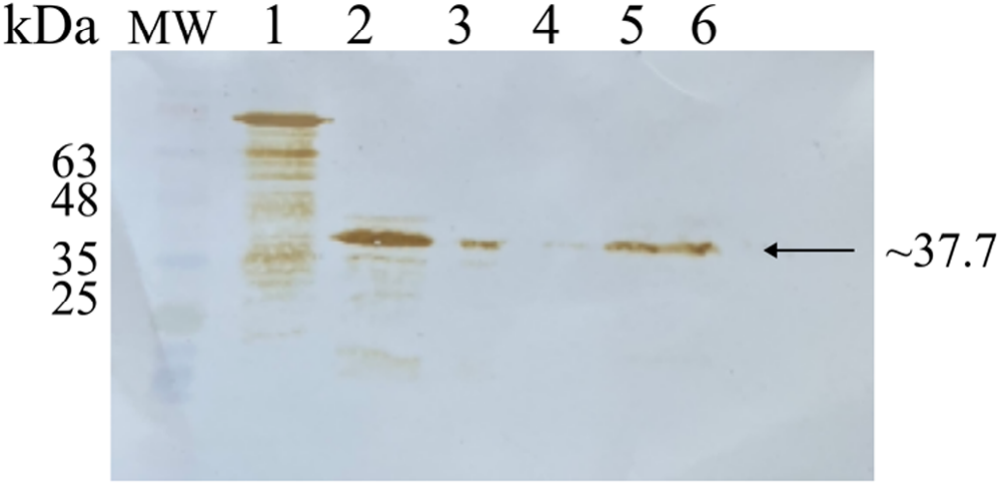
*r*M‐PDCoV different batches purification. The different batches of *r*M‐PDCoV were obtained and confirmed by Western blot. Line 1: a positive control (recombinant viral nucleoprotein of 70 kDa); Line 2: an *r*M‐PDCoV before purification; Lines 2–6: different batches of *r*M‐PDCoV were visualized with a molecular weight of ∼37.7 kDa marked with a black arrow.

Several batches of *r*M‐PDCoV were obtained to develop and apply the iELISA. The *r*M‐PDCoV confirmation was carried out using 12% SDS–PAGE and WB. The protein was visualized with a molecular weight of ∼37.7 kDa (Figure 1).

### 3.2 iELISA Optimization and Analysis of Reference Sera

**FIGURE 2:**
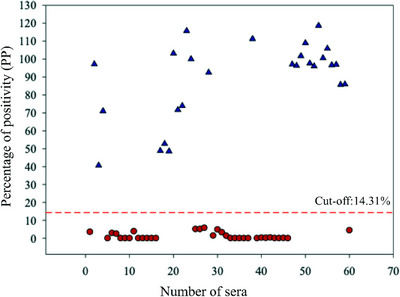
Distribution of the 30 positives (blue triangles) and 30 negatives (red dots) serum sample. Positive and negative samples were selected above or below the cut‐off value (discontinues red line), respectively.

The optimal conditions determined for the iELISA were as follows: 75 ng/well of purified *r*M‐PDCoV; 1:500 of serum sample dilution; 1:15,000 of secondary antibody anti‐pig IgG‐HRP. The highest and lowest PP values for the positive sera were 125.2% PP and 15.2% PP, respectively. The highest and lowest PP values for the negative sera were 13.6% PP and 0.0% PP, respectively. Therefore, the PP values for the negative and positive samples ranged on either side of the cut‐off value of 14.31% (Figure 2). With these conditions, the 516 serum samples were analysed, and the results were expressed as PP (see Section 2).

### 3.3 Specificity, Sensitivity and Concordance (*K*) of iELISA

With the 30 positive and 30 negative sera determined via iELISA and confirmed by WB as a gold standard test, the per cent of specificity, sensitivity and kappa index was determined with a 2 × 2 contingency table (Table 3). We observed that the 30 negatives determined by iELISA were also confirmed negative by WB; this result gives a 100% of specificity. However, six sera determined positive in the iELISA were confirmed negative by WB, giving an 83.33% sensitivity. On the other hand, the kappa index value for the concordance of the test gives a nearly perfect agreement, 0.8831, compared to the WB (see Section 2).

**TABLE 3:** Determination of per cent of sensitivity, specificity and kappa index.

	PDCoV positive	PDCoV negative	Total
Positives serums	24	6	*r = *30
Negative serums	0	30	*s* *=* 30
Total	*t* *= *24	*u = *36	*N = *60
Sensitivity	$\%\;sensitivity=[\;\frac{24}{24+0}\;]\;\times \;100=100\%$		
Specificity	$\%\;specificity=[\;\frac{30}{6+30}\;]\;\times \;100=83.33\%$		
Kappa index	$K=0.9-\frac{0.0166}{1-0.0166}\;Po=\frac{\left(24+30\right)}{60}\;\mathrm{and}\;Pe=\frac{30+24+30+36}{{\left(60\right)}^{2}}$ *K* **= **0.8831		

Abbreviation: PDCoV, porcine deltacoronavirus.

### 3.4 Serological Analysis of PDCoV in Pig Serum Samples

**FIGURE 3:**
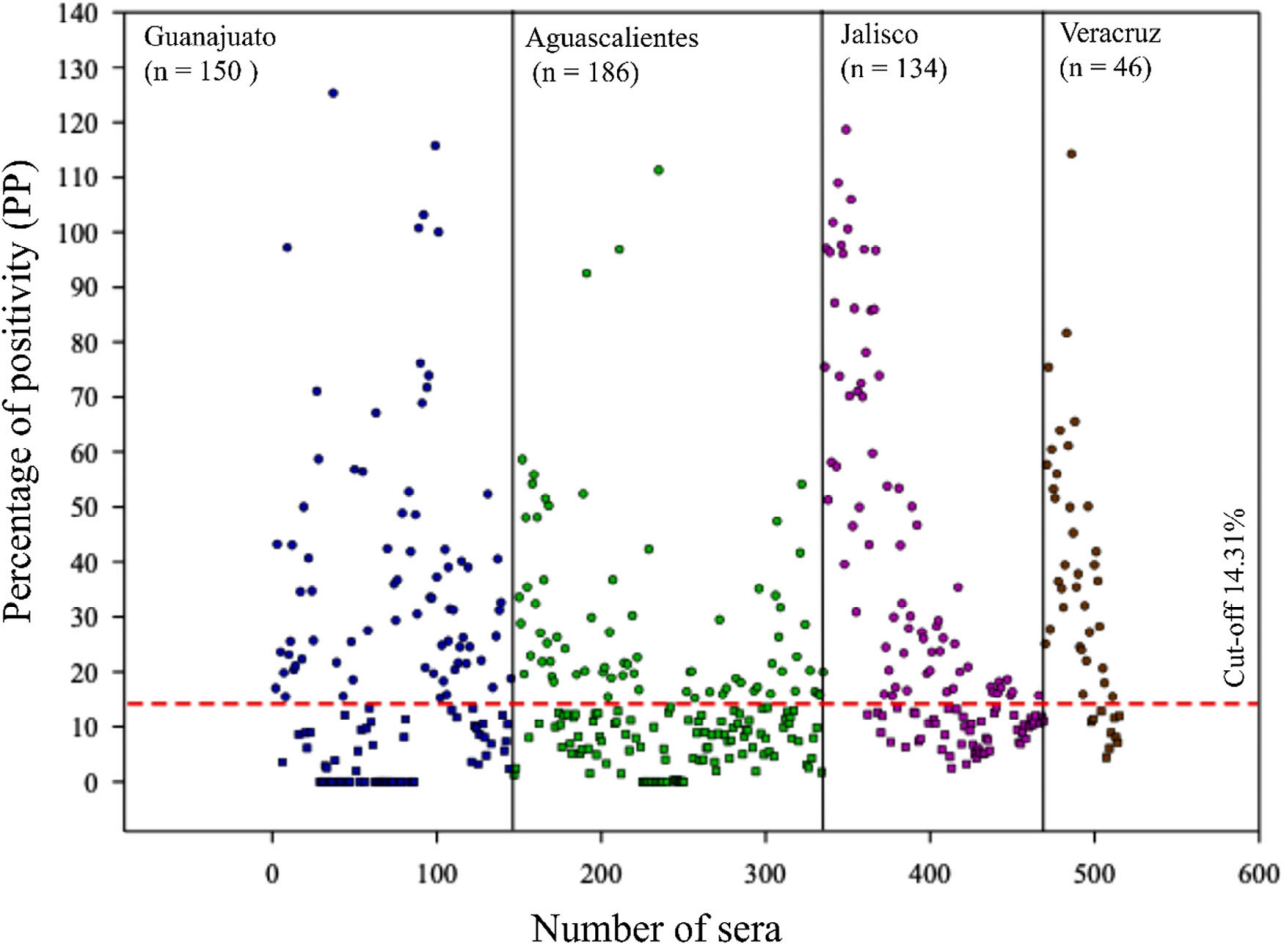
Distribution of the antibody detection by iELISA using 516 sera samples: Guanajuato (blue), Aguascalientes (green), Jalisco (pink) and Veracruz (brown). Positive sera are shown with dots in blue, green, pink and brown, respectively. Negative sera are shown with squares in blue, green, pink and brown, respectively. The cut‐off (14.31%) is shown in a dotted red line.

Using the optimal conditions described above for the iELISA, the serological PDCoV study was determined in 516 pig serum samples from different states in Mexico (Table 4), including 150 sera from Guanajuato, 186 from Aguascalientes, 134 from Jalisco and 46 from Veracruz. The serum samples were collected from 2019 to 2021. Anti‐M‐PDCoV antibodies were detected in 260/516 sera, which represented 50.38% of the samples (Figure 3).

**TABLE 4:** Number of porcine deltacoronavirus (PDCoV) negative and positive serum samples obtained in this study.

	Sera samples (n)	Negative sera	Positives	
			No. positive sera	Percentage (%)
Aguascalientes	186	111	75	40.32
Guanajuato	150	73	77	51.33
Jalisco	134	61	73	54.47
Veracruz	46	11	35	76.08
Total	516	256	260	50.38

Positive serum samples were identified in the four states, with Veracruz showing the highest percentage of positive samples and Aguascalientes showing the lowest percentage of positive samples (Table 4).

**FIGURE 4:**
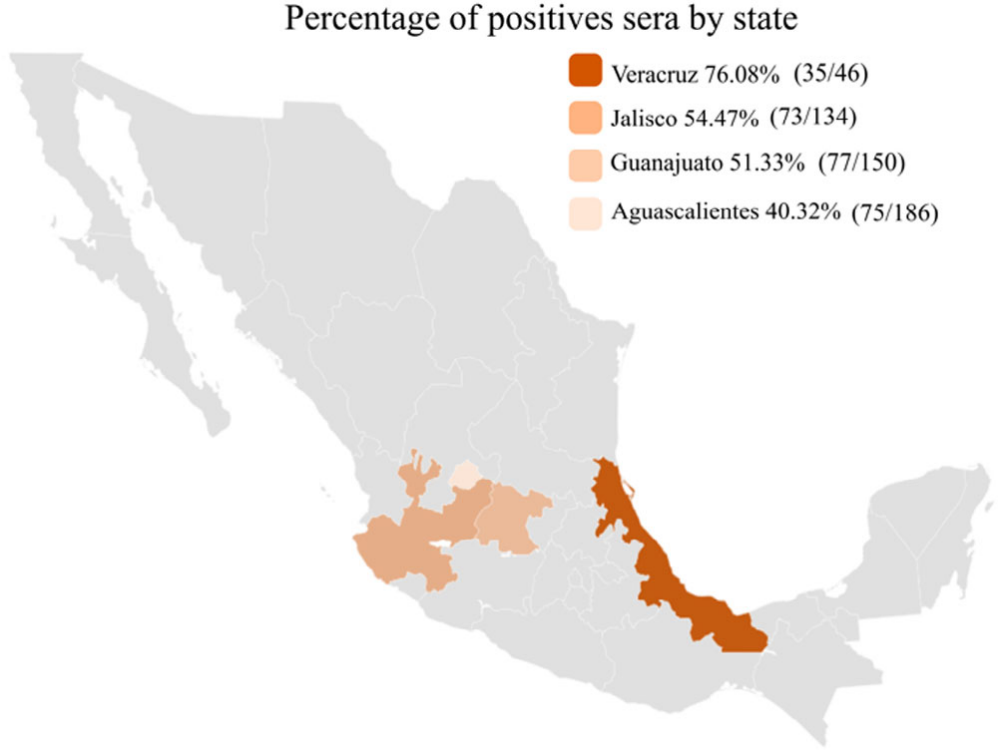
Percentage of positivity sera by state using 516 sera samples. The percentage of positivity is arranged from highest to lowest: Jalisco (54.47%), Veracruz (76.08%), Guanajuato (51.33%) and Aguascalientes (40.32%).

Specifically, we found 75 positive and 111 negative sera in a total of 186 samples from Aguascalientes, which corresponded to a PP of 40.32%. We found 77 positive and 73 negative sera in a total of 150 samples from Guanajuato, which corresponded to a PP of 51.33%. We found 35 positive and 11 negative sera in a total of 46 samples from Veracruz, which corresponded to a PP of 76.08%. Finally, we found 73 positive and 61 negative sera in a total of 134 samples from Jalisco, which corresponded to a PP of 54.47% (Figure 4).

### 3.5 Cross‐Reactivity Determination

**FIGURE 5:**
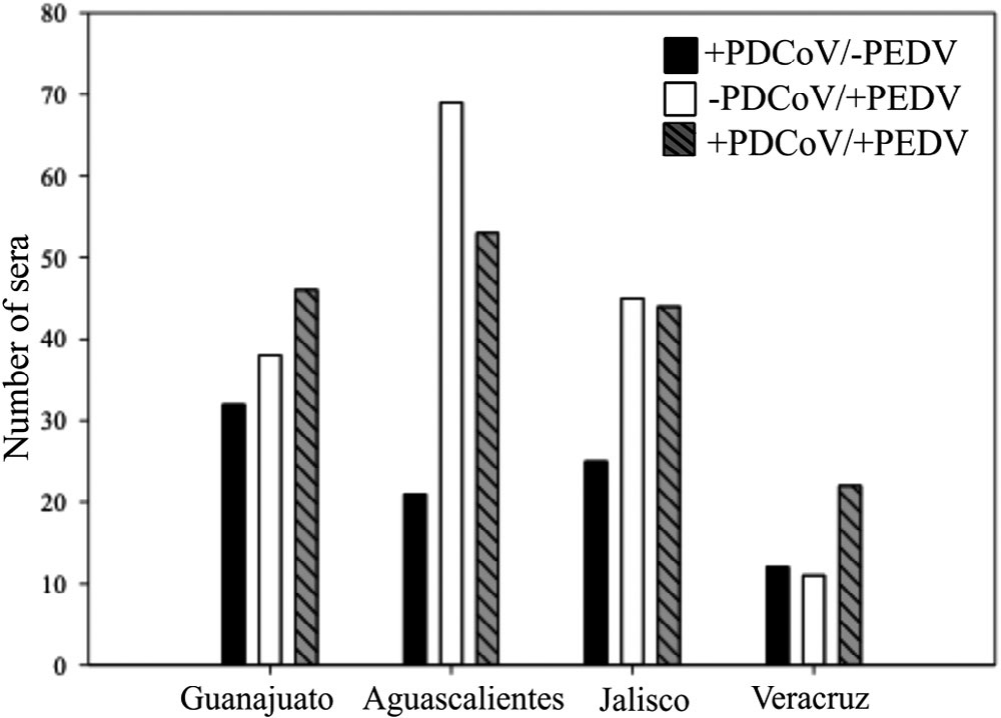
Graph of sera showing samples positive to PDCoV and negative to PEDV (+PDCoV/−PEDV), negative to PDCoV and positive to PEDV (−PDCoV/+PEDV) and positive to PDCoV and positive to PEDV (+PDCoV/+PEDV). Sera samples +PDCoV/−PEDV are shown in black bars, sera −PDCoV/+PEDV are shown in white bars and sera +PDCoV/+PEDV are shown in dark grey striped bars. Veracruz showed the highest number of sera +PDCoV/−PEDV. Aguascalientes showed the highest number of sera −PDCoV/+PEDV. Moreover, Veracruz showed the highest number of sera +PDCoV/+PEDV.

The same 516 serum samples were previously sero‐analysed via iELISA with PEDV recombinant spike protein as an antigen (Garcia‐Gonzalez et al. 2023) with a molecular weight of ∼45 kDa. Therefore, to determine PDCoV–PEDV cross‐reactivity, in this study, we analysed and determined which sera corresponded to single infections for both viruses, PDCoV and PEDV. From a total of 516 samples, we observed 17.44% (90/516) of the sera positive for PDCoV and negative for PEDV (+PDCoV/−PEDV), and 31.59% (163/516) of the sera were negative for PDCoV and positive for PEDV (−PDCoV/+PEDV). On the other hand, from a total of 516 samples, we observed 165/516 (31.98%) sera positive to PDCoV and PEDV (+PDCoV/+PEDV) (Table 5 and Figure 5). To determine whether the single infections were genuine, randomly selected sera samples were analysed using WB to confirm the +PDCoV/−PEDV and −PDCoV/+PEDV sera. Five sera were selected to confirm the −PDCoV/+PEDV sample, and 10 were selected to confirm the +PDCoV/−PEDV sample. Twice sera were selected to confirm the +PDCoV/−PEDV because single infections with PDCoV are not as frequent as PDCoV infections.

**TABLE 5:** Determination of porcine deltacoronavirus (PDCoV)–PEDV cross‐reactivity using the 516 serum samples.

	+PDCoV/−PEDV		−PDCoV/+PEDV		+PDCoV/+PEDV	
	Serum samples	Percentage (%)	Sera samples	Percentage (%)	Sera samples	Percentage (%)
Aguascalientes	21	11.29	69	37.09	53	28.49
Guanajuato	32	21.33	38	25.33	46	30.66
Jalisco	25	18.65	45	33.58	44	32.83
Veracruz	12	26.08	11	23.91	22	47.82
Total	90	17.44	163	31.59	165	31.98

## 4 Discussion

In Mexico, there is no information about the seroprevalence of PDCoV. Therefore, this study aimed to determine the seroprevalence of this disease by developing a specific and sensitive diagnosis system for the effective sero‐valuation of PDCoV infections. It is known that serological assays can quickly detect large numbers of samples with both high sensitivity and high specificity.

To achieve this, 516 backyard pig serum samples from 4 different Mexican states were analysed to develop and validate an iELISA and to determine the seroprevalence of this disease in these four states of Mexico. The iELISA was developed using an *r*M‐PDCoV previously reported by our study group as an antigen (Castaneda‐Montes, Cerriteno‐Sanchez, et al. 2023). Several studies have shown that M protein is a highly conserved protein, so it can be used as an antigen in a serological test like an iELISA (Dong, Zhang, et al. 2016; He et al. 2005; Liu et al. 2010; Qian et al. 2006; Takano et al. 2014; Weiss and Leibowitz 2011; Xing et al. 2009; Zhang et al. 2012). Serological tests can also provide a piece of useful information regarding PDCoV past infections and the seroepidemiology of this disease. Therefore, estimating the number of pigs who have previously been infected with PEDV enables an understanding of how this virus spreads in different locations to evaluate the performance of infectious diseases and enrich our understanding of how this virus spreads.

The iELISA standardization was carried out to determine the optimal conditions by analysing different dilutions of sera and conjugated anti‐pig antibodies. The optimal conditions (antigen 75 ng/well, serum dilution 1:500, and conjugated antibody dilution 1:15,000) were similar to the results obtained by García‐González. E. et al. (antigen 75 ng/well; serum dilution 1:200 and conjugated antibody dilution 1:10,000) for a recombinant NTD‐S protein of a PEDV used in an iELISA (Xing et al. 2009). The sera dilutions used in this study were similar to those used by Su et al. (2016) in an iELISA for a recombinant N‐PDCoV protein. Likewise, the cut‐off value used to discriminate between positive and negative sera samples (0.3732) was similar to the obtained (0.34) by Thachil et al. (2015). On the other hand, it has been mentioned that although iELISAs based on recombinant structural proteins increase specificity, the sensitivity can be reduced due to the heterogenicity of virus isolates (Garcia‐Gonzalez et al. 2023). However, in this study, the percentages of sensitivity and specificity were 83.3% and 100%, respectively, and the concordance (*K*) (0.88) was not affected when using the *r*M‐PDCoV as an antigen. Moreover, for a test to be statistically acceptable, the sum of the sensitivity and specificity values should be at least 1.5 (<1 indicates a useless test, and 2 indicates a perfect test) (Power et al. 2013). We obtained a value of 0.833 for sensitivity and 1.0 for specificity, which gave a sum of 1.833, suggesting an acceptable statistical value support. Therefore, the iELISA developed in this study is suitable for determining the seroprevalence of PDCoV.

In 2105, 8.7% of positive samples were found in different US pig farms (Thachil et al. 2015). Moreover, in 2021, a range of positive samples, 1.28%–41.7%, was found in different pig farms from different regions of China (Tang et al. 2021). The positive samples found in this study, 50.38% (260/516), are higher compared to other studies. This result could be explained because these samples came from backyard pig farms characterized as small‐scale pig sectors with low or no biosecurity controls. It is well known that pig farms with good biosecurity control may reduce the probability of the introduction and spread of pathogens (Alarcon, Allepuz, and Mateu 2021).

We compared the sera positive for PDCoV obtained in this study (50.38%) and the sera positive for PEDV that was previously obtained (62.59%) (Garcia‐Gonzalez et al. 2023). We observed that PDCoV infections (17.44%) were not as frequent as PEDV infections (31.59%). We also observed that coinfections occurred in 31.98% of the sera samples (Table 5). To determine whether the single infections were genuine, randomly selected sera samples were analysed using WB to confirm the +PDCoV/−PEDV and −PDCoV/+PEDV sera. Five sera were selected to confirm the −PDCoV/+PEDV sample, and 10 were selected to confirm the +PDCoV/−PEDV sample. Twice sera were selected to confirm the +PDCoV/−PEDV because single infections with PDCoV are not as frequent as PDCoV infections. All the WB analyses were positive, confirming the infections.

In Mexico, pigs are still traditionally kept in small backyard farms, usually for family needs, and their main characteristics are low biosecurity. This could lead to more infections and a widespread disease, resulting in a high percentage of infected and coinfected pigs (Milicevic et al. 2023).

Overall, our results suggest that PDCoV has been circulating in these regions and that the negative effects have not yet been quantified. Jalisco, Veracruz and Guanajuato are some of the main swine producers. These states represent 40% of national swine production (https://www.gob.mx/siap/acciones‐y‐programas/panorama‐agroalimentario‐258035, accessed March 2024). In this scenario, the effects of PDCoV infections and coinfections could be greater than expected, suggesting a relationship between the seroprevalence of PDCoV and production capacity. The above, emphasizes the need to examine the presence of swine coronavirus and coinfections as well as their impacts on pig farming in Mexico.

## 5 Conclusion

In this study, an iELISA with an *r*M‐PDCoV was validated. The results show that positive and negative sera can be discriminated with high sensitivity and specificity to determine the seroprevalence of PDCoV. Additionally, the iELISA developed in this study can be used in further analyses as an alternative and scalable platform to obtain an official diagnostic method that could be used by government health regulatory institutes. Aditionally, to continually investigate the seroprevalence of PDCoV in Mexico.

## Author Contributions

Francisco Jesus Castañeda Montes and Susana Elisa Mendoza Elvira contributed to the conceptualization. Francisco Jesus Castañeda Montes and José Luis Cerriteño Sánchez contributed to the methodology. Francisco Jesus Castañeda Montes and María Azucena Castañeda Montes contributed to the validation, formal analysis and investigation. Susana Elisa Mendoza Elvira, Julieta Sandra Cuevas Romero and José Luis Cerriteño Sánchez contributed to the resources. Francisco Jesus Castañeda Montes contributed to the writing–original draft preparation. Julieta Sandra Cuevas Romero and José Luis Cerriteño Sánchez contributed to the writing–review and editing. Susana Elisa Mendoza Elvira and Julieta Sandra Cuevas Romero contributed to the visualization, supervision and project administration. Julieta Sandra Cuevas Romero and Susana Elisa Mendoza Elvira contributed to the funding acquisition. All authors have read and agreed to the published version of the manuscript.

## Ethics Statement

The authors confirm that the ethical policies of the journal, as noted on the journal's author guidelines page, have been adhered to and the appropriate ethical review committee approval has been received. This study was carried out following the guidelines of the COMITE DE BIOETICA PARA EL CUIDADO Y USO RAZONABLE DE ANIMALES PARA EXPERIMENTACIÓN EN PROYECTOS DE INVESTIGACIÓN, CENID‐Microbiología Animal; approval number: CBCURAE‐2017/001, approval date: 21 September 2017. Moreover, the experiments were made following the Mexican legislation (NOM‐062‐ZOO‐1999; SAGARPA) based on the Guide for the Care and Use of Laboratory Animals, NRC.

## Conflicts of interest

The authors declare no conflicts of interest.

### Peer Review

The peer review history for this article is available at https://publons.com/publon/10.1002/vms3.70108.

## Data Availability

The authors have nothing to report.
